# Impacts of Cerium Dioxide Nanoparticles on the Soil–Plant System and Their Potential Agricultural Applications

**DOI:** 10.3390/nano15120950

**Published:** 2025-06-19

**Authors:** Nadeesha L. Ukwattage, Zhang Zhiyong

**Affiliations:** 1 Department of Environmental Technology, Faculty of Technology, University of Colombo, Pitipana, Homagama 10206, Sri Lanka; 2 Key Laboratory for Biomedical Effects of Nanomaterials and Nano Safety, Institute of High Energy Physics, Chinese Academy of Sciences, Beijing 100049, China; zhangzhy@ihep.ac.cn; 3 School of Nuclear Science and Technology, University of the Chinese Academy of Sciences, Beijing 100049, China

**Keywords:** cerium dioxide nanoparticles, soil ecosystem, transformations, plant growth, crop production, agricultural applications

## Abstract

Cerium dioxide nanoparticles (CeO_2_-NPs) are increasingly used in various industrial applications, leading to their inevitable release into the environment including the soil ecosystem. In soil, CeO_2_-NPs are taken up by plants, translocated, and accumulated in plant tissues. Within plant tissues, CeO_2_-NPs have been shown to interfere with critical metabolic pathways, which may affect plant health and productivity. Moreover, their presence in soil can influence soil physico-chemical and biological properties, including microbial communities within the rhizosphere, where they can alter microbial physiology, diversity, and enzymatic activities. These interactions raise concerns about the potential disruption of plant–microbe symbiosis essential for plant nutrition and soil health. Despite these challenges, CeO_2_-NPs hold potential as tools for enhancing crop productivity and resilience to stress, such as drought or heavy metal contamination. However, understanding the balance between their beneficial and harmful effects is crucial for their safe application in agriculture. To date, the overall impact of CeO_2_-NPs on soil -plant system and the underlying mechanism remains unclear. Therefore, this review analyses the recent research findings to provide a comprehensive understanding of the fate of CeO_2_-NPs in soil–plant systems and the implications for soil health, plant growth, and agricultural productivity. As the current research is limited by inconsistent findings, often due to variations in experimental conditions, it is essential to study CeO_2_-NPs under more ecologically relevant settings. This review further emphasizes the need for future research to assess the long-term environmental impacts of CeO_2_-NPs in soil–plant systems and to develop guidelines for their responsible use in sustainable agriculture.

## 1. Introduction

Engineered nanoparticles (ENPs) are crucial materials in contemporary science and technology, yet their potential hazards to the environment and biological systems remain a critical concern [1]. Investigating the environmental impacts of ENPs is a pressing scientific priority, as it provides valuable insights into their risks to human health and ecological systems [2]. Among the diverse range of ENPs, cerium dioxide nanoparticles (CeO_2_-NPs) have garnered substantial attention due to their rapidly increasing global production levels—estimated at approximately 10,000 tons annually—and their extensive applications across various industries [3,4]. Commonly referred to as nanoceria, CeO_2_-NPs are nanoscale particles of CeO_2_ that exhibit exceptional structural characteristics, distinctive physicochemical properties, biocompatibility, and environmental stability [5,6]. These attributes contribute to their versatility and render them a highly promising material for numerous advanced technological applications.

### 1.1. Characteristics of CeO_2_-NPs

**Figure 1:**
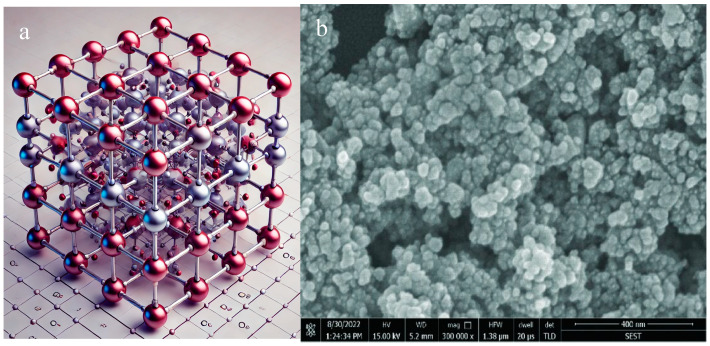
(**a**) A 3D fluorite lattice structure of Ce_4_O_8_ and (**b**) scanning electron microscopic image of CeO_2_-NPs [8].

Typically, CeO_2_-NPs have sizes smaller than 100 nm and can be synthesized in various morphologies, including spheres, rods, cubes, and polyhedrons [1,7]. These nanoparticles generally exhibit high crystallinity, although the degree of crystallinity may vary depending on the synthesis method employed [7]. CeO_2_-NPs predominantly adopt a fluorite crystal structure (CaF_2_-type), characterized by each Ce ion (Ce^4+^) being coordinated with eight oxygen ions in a cubic configuration [8]. However, certain synthesis techniques may result in partially amorphous nanoceria, which can influence its physicochemical properties, stability, and overall performance in applications [5,8]. Figure 1 shows the basic fluorite lattice structure and scanning electron microscopic view of Ce_4_O_8_.

Nano-sized CeO_2_ has oxygen vacancies and defects on the surface, which enables the reversible shift of Ce oxidation states between +3 and +4, depending on the availability of oxygen atoms [9]. The oxidation state Ce^4+^ is predominantly found in CeO_2_, whereas Ce^3+^ is less common, but significant in nanoscale applications [10,11]. The ability to switch between these oxidation states (Ce^4+^ ↔ Ce^3+^) is central to nanoceria’s functionality, as it allows for redox cycling, which is highly valuable in its catalytic and antioxidant applications [12]. Additionally, the nanoscale size of nanoceria gives it a high surface area-to-volume ratio, enhancing its reactivity and emphasizing surface-related properties [13]. For instance, the increased presence of Ce^3+^ ions on the surface of nanoceria, compared to bulk CeO_2_, is tied to the smaller particle size [8]. Functionalizing the surface, such as by coating nanoceria with polymers or other molecules, can further enhance its biocompatibility or catalytic specificity for targeted applications [14].

### 1.2. Environmental Release of CeO_2_-NPs

**Figure 2:**
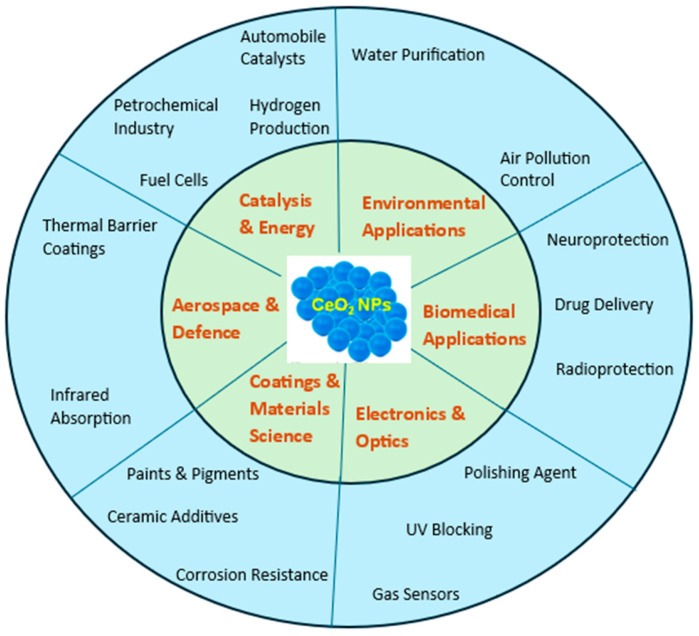
Main applications of CeO_2_-NPs in different industrial sectors.

CeO_2_-NPs are widely utilized in various industrial processes and consumer products, raising concerns about their unintentional release into the environment during manufacturing, usage, and disposal [6]. These nanoparticles can enter air, water, and soil systems via multiple pathways, posing potential risks to ecosystems and contributing to concerns about their long-term accumulation and toxicity [15,16]. Figure 2 illustrates the main applications of CeO_2_-NPs in the industrial sectors.

In the automotive sector, CeO_2_-NPs are employed as fuel additives and in catalytic converters, where they can be emitted into the atmosphere through vehicle exhaust [17]. Industrial high-temperature processes, such as the flame spray pyrolysis used in the synthesis of CeO_2_-NPs, are also significant sources of nanoparticle emissions [5]. Manufacturing facilities producing or utilizing nanoceria in coatings, ceramics, or polishing agents may discharge these particles into the environment via flue gases or wastewater [18].

The antioxidant properties of CeO_2_-NPs have led to their application in medical fields, including in drug delivery systems and diagnostic tools [14]. However, residual nanoparticles from these applications often enter wastewater systems. Conventional wastewater treatment plants typically lack the capacity to effectively filter out nanoparticles, resulting in their release into surface and groundwater systems [19]. Agricultural practices represent another major pathway for introducing CeO_2_-NPs into the soil [16]. Certain fertilizers and pesticides incorporate nanoceria to enhance nutrient delivery or plant protection [20]. The application of such products directly deposits CeO_2_-NPs into the soil, with the potential for subsequent transport to aquatic systems via surface runoff [15]. Additionally, biosolids derived from treated sewage sludge, commonly used as soil amendments, can introduce CeO_2_-NPs into agricultural soils [7].

Consumer products, including sunscreens, cosmetics, polishing agents, textile finishes, and some electronic devices, also contribute to environmental nanoceria contamination [1]. Improper disposal of these products into landfills can lead to soil and groundwater contamination through landfill leachate [9]. Moreover, incineration of nanoceria-containing waste products can release particles into the atmosphere via flue gas emissions. Accidental spills during the production, transportation, or handling of nanoceria pose further risks of contamination, particularly in proximity to natural ecosystems [11]. The widespread use and environmental dispersal of CeO_2_-NPs highlight the need for improved waste management strategies and the development of technologies to mitigate nanoparticle release into ecosystems.

### 1.3. Environmental Fate of Nanoceria

Upon release into the environment, CeO_2_-NPs interact dynamically with various environmental compartments, including air, water, soil, and biota [21]. Their behavior and fate in these matrices are governed by their physicochemical properties (particle size and shape, surface chemistry, zeta potential, oxidation state, hydrophobicity, etc.) and environmental conditions [11]. Owing to their nanoscale dimensions, in air, CeO_2_-NPs can remain suspended as fine particulate matter or aerosols for extended durations [19]. These nanoparticles are prone to aggregation and may undergo chemical transformations influenced by atmospheric conditions such as humidity, temperature, and pollutant interactions [3]. Eventually, they are removed from the atmosphere through gravitational settling or precipitation, leading to their deposition onto terrestrial or aquatic systems [11]. CeO_2_-NPs exhibit low solubility in water but can form stable colloidal suspensions based on the water chemistry, including pH, ionic strength, and the presence of dissolved organic matter [3,22]. Interactions with natural organic matter, sediments, and aquatic organisms can significantly alter the nanoparticles’ surface properties, mobility, and aggregation state [23,24]. Additionally, CeO_2_-NPs display redox activity in aquatic environments, transitioning between Ce^3+^ and Ce^4+^ oxidation states. This redox cycling is influenced by light, pH, and organic matter, which can modulate the particles’ chemical reactivity and toxicity [11,19].

In soil, CeO_2_-NPs interact with soil particles, organic matter, and microbial communities [15,25]. These interactions influence their distribution and persistence. Depending on the soil’s physicochemical characteristics, nanoceria may be adsorbed onto soil constituents or leached into groundwater systems [26]. Despite their strong oxidizing capacity, the chemical stability of CeO_2_-NPs makes them highly resistant to dissolution and degradation, resulting in their long-term persistence and potential accumulation in soil over time [27]. From soil, CeO_2_-NPs can be taken up by plants, microbes, and animals, raising concerns about bioaccumulation and upward movement through the food chain [2,16]. Additionally, ceria nanoparticles can enter plants from air, through foliar uptake, where the particles deposit on the surfaces of leaves and other aerial plant structures [28]. Nanoceria may transfer through food webs, potentially amplifying its ecological impacts at higher trophic levels [11]. The bioavailability of nanoceria is determined by factors such as particle size, surface charge, and the surrounding environmental chemistry [20,27]. The environmental persistence and potential for bioaccumulation of CeO_2_-NPs underscore their ecological significance and the need for further research into their environmental and biological interactions.

## 2. Interaction and Transformations of CeO_2_-NPs in Soil

**Figure 3:**
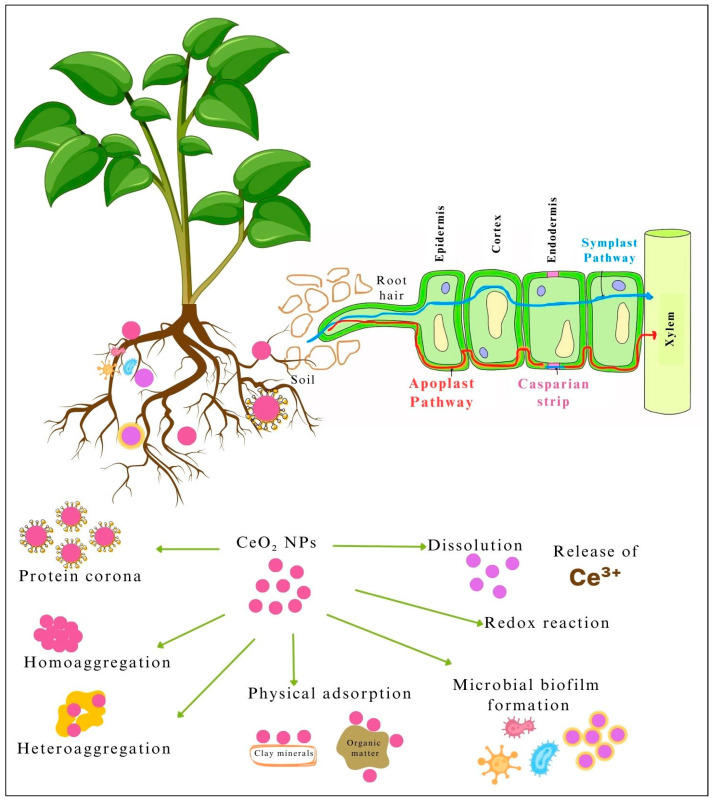
Illustration of the fate of CeO_2_-NPs in soil and root uptake pathways.

Soil ecosystems are the ultimate sink of many nanomaterials, including CeO_2_-NPs [29]. In soil, CeO_2_-NPs either interact with the soil constituents and/or undergo physical chemical and biological transformations. Such interactions and transformations influence the stability, mobility, and bioavailability and thus the potential impacts of CeO_2_-NPs in soil ecosystems [9]. Figure 3 illustrates the fate of CeO_2_-NPs in soil.

Aggregation is the most common physical process, where CeO_2_-NPs form clusters that may settle out of the soil solution, reducing their mobility [30]. Aggregation is primarily driven by van der Waals forces and the development of an electric double layer of counter ions and often influenced by soil ionic strength and pH [22]. In soil, CeO_2_-NPs undergo two primary types of aggregation: homo-aggregation and hetero-aggregation. In homo-aggregation, CeO_2_-NPs cluster together with other similar CeO_2_ particles. In hetero-aggregation, CeO_2_-NPs interact with different soil components, such as clay particles, organic matter, and metal oxides, which leads to the formation of mixed aggregates [29,31]. This form of aggregation is more common in natural soil environments, where a variety of particles are present to interact with the CeO_2_-NPs [32]. Clay is an abundantly present group of soil minerals which often form hetero-aggregates (via sorption) with CeO_2_-NPs, and understanding the chemistry of this process has attracted much recent scientific attention [20,26,33]. CeO_2_-NPs also interacts with phyllosilicates and carbonates present in soil, potentially affecting the subsequent aggregation and mobility [22].

Adsorption is also an important physical process where CeO_2_-NPs are held onto mineral or organic matter in soil. This can either immobilize, prevent leaching, or enhance transportation of nanometric CeO_2_ through soil. The adsorption mechanisms are controlled by several key variables which can be grouped as nanoparticle properties, soil properties, and environmental conditions [26].

The high surface area and large number of active sites present in nanoceria particles enable its strong adsorption onto soil minerals such as clay, quartz, and oxides of iron, aluminum, and manganese [29]. Adsorption mainly occurs through electrostatic attractions between the CeO_2_-NPs and the mineral surfaces [34] and hence is significantly affected by the surface charge and coating of the nanoparticles. Zhang et al. [35] observed a stronger affinity of positively charged CeO_2_-NPs towards Kaolin when compared to negatively charged CeO_2_-NPs. Similarly, adsorption into natural soil organic matter is a predominant transformation mechanism of CeO_2_-NPs that determines its activity in the soil matrix. Organic matter often has negatively charged groups that can attract CeO_2_-NPs through electrostatic interactions [20]. Further, the functional groups in some organic matter, such as carboxyl, hydroxyl, and phenolic groups, can complex with CeO_2_-NPs [33,36]. This type of complexation is stronger and can provide significant stability to CeO_2_-NPs, preventing their aggregation in soil environments. Though CeO_2_-NPs are attracted to negatively charged groups in organic matter, this interaction is strongly dependent on soil pH and ionic strength. The strongest adsorption occurs under acidic to neutral pH conditions where CeO_2_-NPs are positively charged and organic matter is negatively charged [37]. At higher pH, electrostatic repulsion may reduce adsorption, but other mechanisms such as ligand exchange hydrogen bonding and van der Waals interactions may still promote some adsorption. The presence of divalent cations (Ca^2+^ or Mg^2+^) can also act as a bridge between CeO_2_-NPs and organic matter to promote adsorption [22,29]. Further to the above mechanisms, in a protein-rich soil environment (e.g., soils rich with organic matter or root systems) a layer of proteins can be readily adsorbed onto the surface of the CeO_2_-NPs through electrostatic and van der Waals interactions, hydrophobic effects, or covalent bonding [33,35]. This is referred as protein corona formation, which is a critical factor in determining the environmental and biological behavior and ecological and toxicological effects of CeO_2_-NPs in soils.

Dissolution and redox reactions are the primary chemical transformations of CeO_2_-NPs in soil [26,38]. Depending on soil conditions, particularly redox potential and pH, CeO_2_-NPs can undergo redox transformations (transfer of electrons between species). Under reducing conditions, Ce^4+^ in CeO_2_ can be transformed into the more soluble Ce^3+^ form, making it more mobile and bioavailable. As discussed by Pietrzak et al. [39], this transformation is prominent in saturated and/or organic matter-rich soils or sediments. CeO_2_-NPs may partially dissolve in soil, releasing Ce^3+^ into the soil solution. The dissolution is typically slow under neutral to alkaline pH but may increase in acidic soils. According to the findings of Zhang et al. [20], smaller NPs exhibit a higher release of free Ce^3+^ ions compared to the larger particles, while surface coating delays the release. Since smaller CeO_2_-NPs have a larger specific surface area, more surface defects and oxygen vacancies are available. This promotes redox cycling between Ce^4+^ and Ce^3+^, which increases solubility and release into the environment [26,33].

Microbial biofilm formation is a possible biological interaction where some soil microbes form a biofilm on CeO_2_-NPs, altering their surface properties and potentially enhancing or reducing their stability and mobility [40]. Further, microorganisms that produce reducing agents such as organic acids can facilitate the reduction of Ce^4+^ to Ce^3+^, thereby increasing CeO_2_ dissolution [41]. As discussed above, the research findings to date attest the complexity of ceria nanoparticle–soil interactions, which emphasizes the need for comprehensive assessments to understand its implications for soil health.

## 3. Impacts of CeO_2_-NPs on Soil Properties

The interactions between ceria nanoparticles and soil constituents play a crucial role in determining the nanoparticles’ fate, transformations, transport mechanisms, and associated environmental risks. Moreover, these interactions significantly influence the soil’s chemical composition, physical structure, and biological activity [22]. Numerous studies have demonstrated that the magnitude and nature of these effects are strongly dependent on the concentration of nanoparticles and their physicochemical properties, including particle size, shape, surface charge, specific surface area, and surface chemical composition [22,29,33]. Additionally, soil environmental factors such as ionic strength, temperature, pH, porosity, mineral composition, and organic matter content have been shown to modulate these interactions [14,27].

### 3.1. Effects on Soil Chemistry

pH is the most crucial soil chemical parameter which determines nutrient availability, soil health, and plant growth. Many recent scientific endeavors have focused on filling the important knowledge gaps of the soil pH dynamics in the presence of nanoceria in different soil environmental settings [38,42,43]. Accordingly, CeO_2_-NPs have been found to influence the soil pH in a few ways, depending on the soil type, the rate of application, and other environmental conditions. CeO_2_-NPs are often alkaline, and when added to acidic soils, they can mildly increase the pH, moving it closer to neutral [44,45]. Low application rates (e.g., 10–50 mg/kg) have found to be typically effective in acidic soils for pH moderation [45]. Nonetheless, neutral soils experience minimal pH changes even at CeO_2_-NP concentrations above 100 mg/kg. As argued by Dahle et al. [23], in neutral soils, CeO_2_-NPs remain relatively stable with minimal dissolution, leading to minimal pH changes. Hence, CeO_2_-NPs can act as a mild pH buffer; their impact is more pronounced in acidic soils compared to neutral ones [46]. In the already high pH of calcareous soils, ceria nanoparticles show a minimal effect on pH, as a result of the soil’s inherent buffering capacity [24]. However, due to reduced solubility in such soils, CeO_2_-NPs may exhibit limited mobility, potentially immobilizing certain nutrients in localized areas of application.

CeO_2_-NPs can interact with soil P, potentially reducing the P availability to plants. Ce ions (Ce^3+^/Ce^4+^) have a high affinity for phosphate ions, leading to the formation of cerium–phosphate complexes [10], which can reduce the P mobility in the soil. Further, CeO_2_-NPs may reduce the exchangeable K in soil via the mechanisms of competitive adsorption, microbial inhibition, and altered soil pH [47]. These effects are more pronounced in soils with low organic matter content or low cation exchange capacity [48]. Nanoceria also influences N cycling in soil, as it can interact with N species. Microbial N cycling processes may be affected at higher CeO_2_-NP levels in soil, leading to lower N availability over time. However, the overall effect on N dynamics, such as nitrification and denitrification processes, requires further research.

CeO_2_-NPs have the potential to adsorb and chelate heavy metals (e.g., Cd, Pb, As, Cr, and Hg) in the soil, which can affect the mobility and bioavailability of these contaminants. The surface of CeO_2_-NPs often has hydroxyl and carboxyl groups [12] that can attract and hold or make complexes with heavy metal cations, reducing their mobility in the soil. This is particularly effective in acidic soils, where heavy metals are more soluble. Further, the heavy metals can be trapped within the aggregate structures of nanoparticles in the soil, immobilizing the metals and thus preventing them from leaching into groundwater or being absorbed by plants [49]. Conversely, studies have found that CeO_2_-NPs may also mobilize certain metals under specific conditions, increasing their availability and potential toxicity. For instance, the redox transformation of As^3+^ to As^5+^ in the presence of CeO_2_ can enhance the As mobility by creating soluble species [50]. Similarly, the nanoparticle-organic matter interactions can mobilize Cu by forming soluble CeO_2_-organic-metal complexes [22]. This dual role makes it essential to carefully assess the soil environment, CeO_2_ concentration, and application methods when using CeO_2_-NPs in agricultural or remediation settings in soil.

Nanoceria’s ability to undergo redox cycling (between Ce^3+^ and Ce^4+^) allows it to influence the redox potential of soil. It may act as an electron shuttle in the soil, participating in oxidation–reduction reactions, which could affect the redox-sensitive elements (e.g., Fe, Mn, S, As, Cr, Cu, etc.) and compounds (NO_3_^−^, SO_4_^2−^, MnO_2_, etc.) [26]. Further, the redox cycling allows CeO_2_-NPs to act as an antioxidant in soil to scavenge Reactive Oxygen Species (ROS) which might protect soil organic matter from oxidative degradation [36]. However, this redox activity could also lead to the unintended oxidation of organic molecules, which may alter soil organic matter composition [51]. Additionally, CeO_2_-NPs may affect the decomposition of organic matter by inhibiting or enhancing microbial activity involved in organic matter breakdown. Soil organic matter can affect this redox behavior, influencing CeO_2_’s ability to act as a catalyst in redox processes [26].

### 3.2. Effects on Soil Physical Properties

Studies focused on the impacts of CeO_2_-NP incorporation on soil’s physical properties are scarce. Therefore, the authors analyzed available knowledge and findings to logically comprehend the potential effects of ceria nanoparticles on different textured soils. With its extremely fine size, CeO_2_-NPs can either improve or deteriorate soil structure, largely depending on the concentration and the soil texture [52]. In clay-rich soils, the high surface area and reactivity of CeO_2_-NPs can interfere with the electrostatic forces that bind clay particles together. This destabilizes the clay aggregates, leading to dispersion [22]. Dispersed clay particles are more prone to being washed away by water, leading to soil erosion and reduced fertility. Further, the ceria nanoparticles can disrupt natural bonding agents, such as organic matter and metal ions, in soil that contribute to aggregate formation. The dispersion of clay particles and collapse of aggregates due to CeO_2_-NPs [28] may increase soil bulk density and reduce porosity, which hinders aeration, water movement, and microbial activity in soil. Further, the reduced aggregate size can make clay soils more susceptible to compaction, which negatively affects root penetration [53].

In sandy soils, CeO_2_-NPs can improve the structure by acting as a binding agent between soil particles, leading to the formation of micro-aggregates [54], where particles are loosely held together through electrostatic interactions. Enhanced aggregation in soil can lead to the formation of nanoclusters, which can affect the soil’s water retention capacity and hydraulic conductivity by maintaining pore spaces that retain and permeate water. However, the binding of CeO_2_-NPs and sand grains may be limited and may only have a moderate impact unless the soil is supplemented with organic or other soil amendments to increase its cation exchange capacity [52]. The beneficial effects from soil aggregation are mostly noticeable in medium-textured loamy soils where a balance of sand, silt, and clay allows the nanoparticles to support natural aggregation processes without risking destabilization.

### 3.3. Effect on Soil Microbiology

CeO_2_-NPs can alter the rhizosphere microenvironment, which directly and indirectly affects the microbial diversity, abundance, metabolic activity, and functional roles within soil ecosystems [38]. The interaction of nanometric CeO_2_ with soil microbial communities is complex. The nanomaterial exhibits selective toxicity, where sensitive microbial and fungal species are inhibited or killed, while others might survive or even proliferate [45]. This can alter the composition of the microbial communities and disrupt the balance between beneficial and pathogenic microorganisms in the soil ecosystem. For example, CeO_2_-NPs have been shown to reduce the abundance of ammonia-oxidizing bacteria, which play a key role in N cycling, leading to altered nutrient dynamics in soil [39].

As discussed by Wang et al. [55], the toxic effects of CeO_2_-NPs on soil microbes occur mainly through two mechanisms: oxidative stress and cell membrane disruption. Nanoparticles may induce oxidative stress in soil microbes with their redox properties. The redox cycling between Ce^3+^ and Ce^4+^ in CeO_2_-NPs produces ROS, such as hydroxyl radicals and superoxide anions [13]. These ROS cause oxidative damage to microbial cellular components, including lipids, proteins, and DNA, potentially leading to cell death. For example, studies have shown that *Escherichia coli* and *Pseudomonas aeruginosa* exposed to CeO_2_-NPs experience increased ROS levels [56], resulting in significant oxidative stress and reduced cell viability. In addition, nanoceria can interact with microbial cell membranes, disrupting their integrity. The nanoparticles can adhere to and penetrate through the lipid bilayer, compromising its structure. This leads to increased membrane permeability, leakage of cellular contents, and ultimately, cell lysis [57]. For instance, *Bacillus subtilis* exposed to CeO_2_-NPs exhibited disrupted cell membrane integrity, which led to the leakage of intracellular contents and cell death [58]. Further to the above mechanisms, genotoxic effects of CeO_2_-NPs on soil microbes by inducing DNA damage have also been discovered [59]. According to recent findings, exposure to CeO_2_-NPs can lead to DNA fragmentation and mutations in bacterial cells, affecting their growth and reproduction [60]. Studies on *Escherichia coli* and *Pseudomonas putida* have demonstrated DNA damage in cells exposed to CeO_2_, which could have long-term consequences on microbial population stability and soil health. Additionally, CeO_2_-NPs have been found to interfere with quorum sensing (the chemical communication process of microorganisms) by adsorbing signaling molecules or by inducing oxidative damage to signaling pathways [38,58]

In addition to the effects on the abundance and diversity of microbial communities, CeO_2_-NPs in the rhizosphere environment can significantly hinder microbial activities and functional roles in soil plant systems. Soil microbes produce extracellular enzymes to break down complex organic compounds in root exudates into smaller, more digestible molecules. CeO_2_-NPs can inhibit the activity of these enzymes by binding to them or by generating ROS, which inactivate the enzymes. Research has shown that CeO_2_-NPs reduce the activity of cellulases and proteases, which are necessary for breaking down carbohydrates and proteins in root exudates [26], possibly leading to an accumulation of unused organic compounds or altered nutrient availability for plants.

The presence of CeO_2_-NPs in soil can impair soil enzymatic activities, which are crucial for nutrient cycling and organic matter decomposition. For example, enzymes involved in C cycling, such as cellulases and β-glucosidases (which help convert complex carbohydrates into glucose), often show reduced activity in the presence of CeO_2_-NPs due to oxidative damage [24]. Enzymes involved in N cycling, such as urease, nitrate reductase, and nitrite reductase are also inhibited by CeO_2_-NPs, reducing nitrogen availability to plants. These enzymes are essential for ammonification, nitrification, and denitrification processes, which convert N between its various forms. CeO_2_-NPs reduce the activity of phosphatase enzymes [61], which play a vital role in releasing inorganic P from organic compounds, making it available for plant uptake. In addition, the redox properties of nanoceria may affect the activity of oxidative enzymes such as peroxidases, involve in organic matter decomposition [6]. Furthermore, CeO_2_-NPs can influence microbial biofilm formation, potentially altering how soil microbes interact with soil particles and organic matter. Since CeO_2_-NPs can harm the survival of soil microbes, the overall production and activity of microbial enzymes are reduced [45]. Decreased production of extracellular enzymes leads to impaired soil nutrient dynamics and plant health.

### 3.4. Effects on Plant Microbial Symbiosis

Symbiotic relationships between plants and soil microorganisms are essential for nutrient acquisition, plant growth, and resilience. By affecting microbial communities in multiple ways, CeO_2_-NPs alter the symbiotic relationships between plants and beneficial microorganisms. The impact on the symbiotic relationship between rhizobia bacteria and leguminous plants is a classic example of this. CeO_2_-NPs induce oxidative stress in rhizobia cells, reducing their viability and activity in the rhizosphere [62]. Additionally, nanoceria can interfere with the signaling between rhizobia and plant roots, inhibiting root nodulation. Studies have shown that plants exposed to CeO_2_-NPs form fewer and smaller nodules, which decreases N fixation efficiency in plants [63]. A mutualistic relationship with mycorrhizal fungi helps in extending the plant root system and enhancing water and nutrient uptake (especially P) in many plant species. In the presence of CeO_2_-NPs, reduced colonization rates of mycorrhizal fungi on plant roots have been observed, mainly due to CeO_2_-NPs’ toxic effects on the fungal spores or hyphae [62].

CeO_2_-NPs inhibit the metabolic activities of plant growth-promoting rhizobacteria, such as *Pseudomonas*, *Azospirillum*, and *Bacillus* species, which enhance plant growth by producing growth hormones, solubilizing nutrients, and protecting against pathogens [64]. Moreover, by limiting nutrient availability in the rhizosphere, CeO_2_-NPs can disrupt the nutrient solubilization functions of these bacteria. Further, CeO_2_-NPs can reduce the viability of this beneficial group of bacteria, which protects plants against diseases, by producing antibiotics or by competing with pathogens [31]. Consequently, the weakening of the natural defense system of the plants may make them more vulnerable to disease. Symbiotic relationships rely heavily on chemical signaling for recognition, attachment, and colonization. CeO_2_-NPs may adsorb or interfere with these signaling molecules (e.g., flavonoids for rhizobia–legume signaling), disrupting the initial stages of symbiotic associations [65]. When signaling is compromised, microbial partners may not establish effective symbiosis with plants, reducing mutual benefits.

The extent to which CeO_2_-NPs can impact on plant–microbe symbiosis depends on factors such as nanoparticle size, concentration, soil environment, and plant species. In their experiment, Ge et al. [66] applied CeO_2_-NPs into planted and unplanted soils and observed no impacts on bacterial communities in unplanted soils at a 100 mg kg^−1^ rate. However, an alteration of soil bacterial communities was observed when soybean plants were present. This important finding linked low CeO_2_-NPs doses to potential changes in the quantity and composition of plant root exudates, which then interactively promoted effects to microbes in the soil. Additionally, when canola plants were grown for 30 days in soil spiked with three differently designed CeO_2_-NPs at 1 mg kg^−1^ at the time of planting, an impact gradient on microbial activity and bacterial community structure was identified, with maximum effects near the root surface compared to the rhizosphere or bulk soil [42]. In other experiments run without plants, 42-day soil exposures to CeO_2_-NPs were reported to have limited impacts on bacterial community structures [67]. This observation highlights the role of plants in promoting interaction between soil microbes and CeO_2_-NPs. Further, studies have revealed that short-term exposure of soil to nanoparticles may have transient effects, while long-term exposure can lead to chronic impacts on microbial health and plant–microbe interactions [64,68]. The effects of CeO_2_-NPs on critical interactions between plants and bacterial communities in the root, rhizoplane, and outer rhizosphere compartments are not well understood, particularly for important crop species.

## 4. CeO_2_-NP Plant Uptake, Translocation, and Transformation

CeO_2_-NPs enter plants mainly through the roots, which act as the primary interface between the plant and the soil. The root uptake can happen through two key routes: apoplastic and symplastic [26]. Figure 3 illustrates the main routes of nanoceria entrance into the plant root. In the apoplastic pathway, CeO_2_-NPs can either enter through the cell wall or intercellular spaces. Movement of CeO_2_-NPs through the cell wall matrix is limited by the size and structure of cell wall pores, which can range from 5–20 nanometers [39]. Smaller CeO_2_-NPs are more likely to move freely in the apoplast, while larger ones may be partially blocked or restricted. Entry through the root epidermis, where CeO_2_-NPs penetrate the root surface by moving along the apoplast, is the most direct pathway for nanoparticle uptake and does not require the nanoparticles to cross any membranes. In the symplastic pathway, nanoceria is taken up into plant cells by passing through the plasma membrane. Some CeO_2_-NPs can enter root cells through endocytosis, a process where the cell membrane engulfs particles, forming vesicles that carry the nanoparticles into the cell [47]. This mechanism allows nanoparticles to bypass cell walls and enter the symplast, or the inner cellular space. Symplastic transport of smallest nanoparticles can also occur through plasmodesmata [69]. Symplastic transport navigates barriers such as the Casparian strip [70]. The apoplastic pathway is essential for radial movement, allowing nanoparticles to reach the root’s central cylinder and vascular tissues. In addition to the above mechanisms, organic compounds in root exudates may chelate small Ce nanoparticles, enhancing their solubility to allow entering the root cells through passive diffusion or through transport proteins that typically transport metal ions. However, most nanoparticles are too large for direct transport by ion channels, so this is generally limited to smaller particles or ions released from NPs [20].

Once CeO_2_-NPs enter the plant root system, they can be translocated to other parts of the plant, including the shoots, leaves, and possibly the fruits or seeds. Understanding how nanoparticles move within plants is crucial for predicting their distribution and accumulation. Ceria nanoparticles predominantly move through the xylem vessels with the transpiration stream [26]. The translocation through the xylem depends on the particle size and the plant’s transpiration rate. Studies suggest that phloem transport may also occur, enabling movement from shoots to roots or other parts of the plant. This implies the potential possibility of foliar-deposited CeO_2_-NPs to enter the plant tissues [71]. However, the phloem transport of nanoceria is less efficient and less understood to date, compared to xylem transport.

Nanoceria tends to accumulate heavily in the root tissues, as they are the primary site of uptake. The nanoparticles may be retained in the root cell walls, vacuoles, or apoplastic spaces [70]. When translocated to the shoots, CeO_2_-NPs are often localized in the leaf epidermis, vascular bundles, or mesophyll cells. In the leaves, nanoceria can remain trapped in specific tissues, including chloroplasts, or be excreted onto the leaf surface via guttation [39]. In some cases, nanoceria may reach the reproductive organs, but the extent of this translocation is species-dependent and may be minimal compared to that in roots and shoots. The accumulated nanoparticles are found to be strategically utilized within plant tissues; for instance, delivering plant hormones during flower development or herbicides targeting parasitic plants [20,27].

Rapid upward movement of the xylem sap limits the interaction of nanoparticles with sap components, whereas in the phloem, the slower flow allows for potential interactions [72]. Within plant tissues, CeO_2_-NPs undergo a range of transformations, including redox reactions, dissolution and ion release, complexation with organic molecules, re-precipitation as Ce compounds, interaction with antioxidants, and aggregation. These transformations are heavily influenced by the plant’s internal environment and help determine the fate, mobility, and potential impact of CeO_2_-NPs on plant physiology. While the presence of CeO_2_-NPs and their transformed species in plant tissues has been reported in many studies, the mechanisms driving these transformations remain largely unexplored to date.

Recent studies have revealed that plant uptake, translocation, and transformation of CeO_2_-NPs differ significantly from other commonly studied metal oxide NPs. For example, ZnO and CuO NPs are more prone to dissolution in the rhizosphere, leading to greater ion release and systemic mobility in plants compared to CeO_2_-NPs, which remain largely intact or undergo limited transformation [4,7]. TiO_2_-NPs, while more stable, are typically less bioavailable due to their low solubility and tendency to aggregate. In contrast, CeO_2_-NPs exhibit moderate mobility, often accumulating in roots but showing limited translocation to aerial tissues unless applied at high concentrations or under stress conditions [39,73]. Compared to Fe_3_O_4_-NPs, CeO_2_-NPs are more likely to undergo redox transformations (Ce^3+^/Ce^4+^), enabling ROS scavenging, whereas Fe_3_O_4_ mainly influences iron nutrition through ion release. Thus, CeO_2_-NPs exhibit unique redox-based antioxidant functions inside plant tissues not typically observed with other metal oxide NPs [25,74].

## 5. Effects of CeO_2_-NPs on Crop Plants

CeO_2_-NPs have shown both positive and negative effects on crop plants through their direct and indirect impacts on seed germination, plant morphology, plant physiology, antioxidant enzymes, gene expression, and crop nutritional value [20,65,69,75].

### 5.1. Impacts on Seed Germination and Seedling Growth

Thus far, the studies focused on the effects of CeO_2_-NPs on seed germination and seedling growth have yielded inconsistent results. Numerous researchers have reported adverse impacts on germination rates and early seedling development in crop plants exposed to CeO_2_-NPs. In contrast, some studies have found no significant effects, while a few have documented enhanced germination and improved seedling growth in the presence of nanoceria. An analysis of the available literature suggests that the observed discrepancies are primarily influenced by two critical factors: plant species and the applied nanoparticle dosage.

For instance, López-Moreno et al. [76,77] reported reductions in seed germination rates for *Cucurbita pepo*, *Zea mays*, and *Solanum lycopersicum* by 20%, 30%, and 30%, respectively, compared to the control, following exposure to CeO_2_-NPs at a concentration of 2000 mg/L. In contrast, the germination rate of *Medicago sativa* seeds remained unaffected under the same exposure conditions. Conversely, Yang et al. [78] observed enhanced seed germination in the medicinal subshrub species *Vitex negundo* at a CeO_2_-NPs concentration of 500 mg/L. However, inhibitory effects were noted at lower (1 mg/L) and medium (100 mg/L) concentrations. Interestingly, their findings on seedling growth revealed responses opposite to those observed for germination. While many studies have reported a concentration-dependent influence of nanoceria on seed germination and early plant development, Anderson et al. [79], in their experiment involving ten plant species (*Allium cepa*, *Avena sativa*, *Brassica oleracea capitata*, *Crocus sativus*, *Daucus carota*, *Glycine max*, *Lactuca sativa*, *Lolium perenne*, *Lycopersicon esculentum*, and *Zea mays*) were unable to establish a conclusive correlation between their observations and the mass-based exposure concentrations of CeO_2_-NPs (0 μg/mL, 250 μg/mL, 500 μg/mL, and 1000 μg/mL) used in the media.

In another study, Zhao et al. [80] investigated the effects of CeO_2_-NPs on *Zea mays* at concentrations of 400 and 800 mg/kg and found no significant influence on seed germination rates. Similarly, Wang et al. [81] reported no effects on germination rates or leaf development in hydroponically grown tomatoes (*Solanum lycopersicum* L.) at CeO_2_-NP concentrations of 0.1, 1, and 10 mg/L. Further evidence supporting the lack of influence of CeO_2_-NPs on seed germination was provided by Mattiello et al. [82]. Under their experimental conditions, the germination of *Hordeum vulgare* L. (barley) remained unaffected even at the highest tested concentration of nano CeO_2_ (2000 mg/L). However, notable effects were observed at the seedling stage. Specifically, seedlings exposed to lower concentrations of CeO_2_-NPs exhibited a significant reduction in root elongation compared to the control group.

### 5.2. Impacts on Crop Plant Physiology

Photosynthesis is the key physiological process of plants that determines its growth and yield. Studies have shown both promoted and hindered photosynthetic activity in plants treated with CeO_2_-NPs in diverse environmental settings. In some crop species, up to a threshold level (governed by plant and environmental factors), nanoceria supplementation enhances chlorophyll synthesis and thus photosynthetic efficiency. This is mainly due to the role of Ce ions in promoting Fe uptake and chlorophyll biosynthesis and enhancing overall plant vigor [39]. Further, CeO_2_-NPs enhance the activity of the rubisco enzyme, which catalyzes CO_2_ fixation in the light-independent phase of the photosynthesis reaction. However, excessive exposure to nanoparticles may lead to oxidative stress, damaging chlorophyll molecules, disrupting enzyme functions, and ultimately inhibiting photosynthetic efficiency. For instance, Abbas et al. [83], in their study with hydroponically grown wheat (*Triticum aestivum* L.), observed a dual role of CeO_2_-NPs, showing positive effects at low doses and toxicity at higher concentrations. Up to 500 mg/L of nanometric CeO_2_ positively influenced the net photosynthesis rate in wheat plants by enhancing stomatal conductance and activating physiological processes. These benefits were linked to the activation of heat shock proteins and the antioxidative enzyme-mimicking properties of CeO_2_-NPs. Conversely, the tested high concentration (2000 mg/L) caused excessive ROS production, leading to oxidative stress and structural damage to the stomata, thereby inhibiting photosynthesis. Skiba et al. [84] also detected positive effects of CeO_2_-NPs on photosynthesis in hydroponic pea plants (*Lathyrus oleraceus* Lam.). Application of 100 mg/L nanoceria resulted in a 40% increase in net photosynthesis, a 36% improvement in stomatal conductance, and a 30% enhancement in water use efficiency compared to the control plants.

Some researchers have detected a reduction in plant chlorophyll content without having impact on net photosynthesis. For example, Zhang et al. [85] reported 16% reduction in relative chlorophyll content in radish plants (*Raphanus raphanistrum* subsp. *sativus* L.) grown in aqueous medium with CeO_2-_NPs at 10 mg/L because of impaired root uptake of the Mg and Fe required for the synthesis of green pigments. However, the mechanical efficiency of the photosystem has not been impacted. Similarly, Priester et al. [66] noticed a decrease in chlorophyll level without having effects on photosynthetic parameters in soybean (*Glycine max* L.) grown in soil treated with CeO_2_-NPs at concentrations of 100, 500, and 1000 mg/kg, respectively. Interestingly, the observed decrease in chlorophyll a and chlorophyll b levels was not proportional to Ce concentration in the growth medium. Such dose-independent impacts on the leaf chlorophyl contents and the photosynthetic efficiency are evident in some other studies. The observed inhibition of chlorophyll a and chlorophyll b synthesis by Bandyopadhyay et al. [86] in the leaves of alfalfa (*Medicago sativa* L.) grown in soil treated with CeO_2_-NPs at concentrations of 250, 500, and 750 mg/kg was not correlated with the dose of the nanoparticles in the growth medium.

Under stress conditions, such as in drought, heat, heavy metal toxicity, or salinity, the excessive levels of ROS production in plant cells can result in a secondary stress called oxidative stress, which damages cellular structures and impairs photosynthesis [87]. CeO_2_-NPs help in scavenging ROS produced in plant cells with its remarkable redox homeostasis, modulated by the activity of antioxidant enzymes such as superoxide dismutase, catalase, and peroxidase. This enhances stress tolerance in crop plants and sustains metabolic functions, and thus resilience. Yang et al. [75] observed significantly high production of H_2_O_2_ in plant tissues compared to the control, in Thale cress (*Arabidopsis thaliana* L.) exposed to nanometric CeO_2_ at concentrations of 100, 200, 500, 1000, and 2000 mg/L. Further, enhanced antioxidant capacity through increased catalase and ascorbate peroxidase activity has been demonstrated in radish (*Raphanus raphanistrum* subsp. *sativus* L.) grown in the traditional soil method in contact with CeO_2_-NPs at 62.5 to 500 mg/kg [88].

Protection of plants from the heat-induced generation of ROS by stabilizing cellular membranes and enhancing heat shock protein activity by CeO_2_-NPs have been reported [66]. Under drought conditions, CeO_2_-NPs help enhance the water-use efficiency of plants by modulating stomatal behavior. Reduced stomatal closure allows plants to maintain gas exchange and photosynthesis, thereby reducing the impact of drought on plant growth. Under salt stress, CeO_2_-NPs can reduce ionic toxicity by decreasing sodium uptake and supporting potassium retention. This regulation of ion balance helps maintain cellular stability and prevents the osmotic and oxidative stress commonly associated with salinity [89]. CeO_2_-NPs have also shown potential in reducing damage from pollutants, as they can adsorb and deactivate various pollutants, reducing their direct toxicity to plants [90].

However, at higher concentrations, nanoceria can trigger oxidative stress in plants by inducing overproduction of ROS in plant tissues. This pro-oxidant effect may overwhelm the plant’s natural antioxidant defenses, leading to accumulation of H_2_O_2_, particularly in roots, lipid peroxidation, and membrane damage and disruption of cellular homeostasis and metabolic functions. The ability of CeO_2_-NPs to generate ROS in plant tissues was confirmed in soybean (*Glycine max* L.) by Priester et al. [66]. Xu et al. [91] reported elevated production of ROS, and thus induced oxidative stress, triggered by CeO_2_-NP exposure in wheat (*Triticum aestivum*) plants, which resulted in damaged cellular membranes. Similarly, Gui et al. [46] observed a significant decrease in the enzymatic activity of superoxide peroxidase and dismutase in the roots of lettuce (*Lactuca sativa* L.) treated with 1000 mg/kg of CeO_2_-NPs. The authors determined a high level of malondialdehyde in the roots, which may indicate damage to the cell membrane caused by ROS. However, the tested lower concentrations (50 and 100 mg/kg) did not have a significant impact on the activity of the enzymes. Consequently, the ability of nanometric CeO_2_ to either alleviate or induce oxidative stress highlights the importance of understanding the dose–response relationships.

### 5.3. Impacts on Plant Growth and Yield

CeO_2_-NPs can have varying effects on crop growth and yield, influenced by factors such plant species, dosage, experimental setup, growth media, and exposure duration. According to past researchers, the influence of CeO_2_-NPs on plant growth and biomass production is closely related to the concentration of nanoparticles in the growing media (soil, potting mix or nutrient solution) and is dependent on the type of crop. It is commonly known that low concentrations of rare earth elements, including Ce, have a positive impact on plant growth and yield, but can result in toxicity at higher concentrations [20,27,39]

Nanoceria can stimulate root elongation and shoot growth and branching by improving the availability of essential macro- and micronutrients such as N, P, K, Mg, and Fe through numerous mechanisms discussed in previous sections of this article. Further, nanometric CeO_2_ in growth media can help mitigate oxidative stress in root tissues and promote cell division and elongation in the root apical meristem. A positive influence of low levels of CeO_2_-NPs on plant hormones, such as auxins, cytokinin, and gibberellins, which regulate cell division and elongation in roots and contribute to shoot growth has been observed [92]. Increased root growth can improve water and nutrient uptake, while healthy shoot growth contributes to overall biomass. Further, the influence of nonmetric CeO_2_ on plant hormonal pathways is crucial for floral induction and flower development in crop plants. By boosting photosynthesis and increasing stress and disease tolerance, nanoceria can enhance the yield of crops. However, such effects are highly concentration and species dependent.

For instance, Priester et al. [93] reported a yield reduction of 22.5% in soybean plants at a CeO_2_-NP concentration of 1000 mg/kg. Similarly, Wang et al. [94] observed CeO_2_-NPs induced significant inhibitory effects on plant growth of paddy at exposure concentrations of 500 mg/kg. Interestingly, other studies have observed no phytotoxic effects on *Cucumis sativus* when exposed to concentrations as high as 2000 mg/kg in Hoagland solution [95]. Furthermore, at a dose of 100 mg/kg, CeO_2_-NPs demonstrated beneficial effects, enhancing photosynthesis and growth in *Lactuca sativa* [46]. At a concentration of 200 mg/kg, CeO_2_-NPs were shown to decrease the photosynthetic rate and CO_2_ assimilation efficiency in *Clarkia unguiculata* [96]. Table 1 summarizes some of the prominent results on the growth and yield responses of crop plants under nanoceria treatment in different experimental settings.

Different plants respond differently to CeO_2_-NPs in a dose-dependent manner and some plants exhibit phytotoxic effects to CeO_2_-NPs even at low dosages. Hence, understanding the response of crops to the presence of nanoceria and the underlying mechanism of intoxication is crucial for their application, especially in food crops. With this regard, assessment of the presence of Ce ions in edible parts of the crops have gained recent scientific attention. Wang et al. [81], in their study with tomato plants treated with CeO_2_-NPs at 0.1–10 mg/L rates, confirmed the translocation of Ce ions into above-ground tissues, including the fruits. Further, Zhang et al. [85] observed the accumulation of Ce in radish plant tissues at a 10 mg/L nanoceria application rate. The mode of application (e.g., soil amendment, foliar spray, or seed treatment) also seems to have an influence on how effectively nanoceria interacts with the plant and rhizosphere, impacting yield outcomes. Notably, many previous studies were carried out in controlled environments, including in green house settings, for a short duration of time and with root application of CeO_2_-NPs. Therefore, long-term studies should explore how nanoceria affects the entire plant growth and development process, especially under field conditions.

### 5.4. Impacts on Crop Nutritional Quality

Root application of nanoceria can influence the uptake of essential nutrients in plants, which directly impacts crop nutritional quality. By improving root development and increasing the bioavailability of nutrients in the soil, CeO_2_-NPs can enhance plant nutrient uptake. This is particularly beneficial in nutrient-poor or degraded soils. Further, nanoceria can improve the levels of antioxidants, secondary metabolites, proteins, and vitamins in crops, contributing to improved nutritional quality. Rossi et al. [105] detected increased Mg accumulation in leaves and prompted higher chlorophyll levels in canola (*Brassica napus* L.) plants treated with nanoceria at the levels of 200 and 1000 mg/kg. Soybean (*Glycine max*) plants treated with CeO_2_-NPs at 0–1000 mg/kg concentrations showed high Cu and P contents in the plant tissues, indicating improved plant nutritional values [106].

Despite these results, many researchers have observed a disrupted uptake of essential plant nutrients in the presence of nanoceria in growth media due to competition and/or altered transport mechanisms. For example, Corral-Diaz et al. [88] observed a significantly reduced accumulation of S in the roots of radish plants treated with Ce at doses of 62.5, 125, 250, and 500 mg/kg. As suggested by the authors, this may be due to the blocking of the uptake of sulfur in the form of SO_4_^2−^ ions, caused by the formation of Ce (SO_4_) in the soil solution. Similarly, a 500 mg/kg CeO_2_-NP treatment negatively affected the nutritional composition of barley kernels. The amylose, K, and S content in the kernels were reduced, whereas β-glucan content was unaffected [107]. Reduced Fe uptake by plants treated with nanometric CeO_2_ is also reported due to competition between Ce^3+^ and Fe^3+^ [97]. Further, studies have observed reduced S, prolamin, glutelin, lauric and valeric acids, and starch in rice grains supplemented with CeO_2_-NPs at 500 mg/kg. In a study by Gui et al. [108], CeO_2_-NPs showed no adverse effects on the contents of organic and mineral nutrients at an environmentally relevant concentration (25 mg/kg). However, at higher concentrations (75 and 225 mg/kg), CeO_2_-NPs significantly changed the nutritional quality of corn kernels and soybean seeds in a species-dependent manner. The effect on the nutritional composition of corn kernels is mainly in organic components, while that of soybean seeds is mainly in mineral elements. Overaccumulation of nanoceria in plant tissues can lead to toxicity, impairing enzymatic functions and disrupting protein synthesis and carbohydrate metabolism. However, the implication of the accumulation of Ce metal elements in plants is yet to be determined [26].

## 6. Applications of CeO_2_-NPs in Agriculture

In recently years, nano agrochemicals, mainly nano pesticides and nano fertilizers, have been successfully applied in crop agriculture, with 20–30% higher efficacy than their conventional products [109]. CeO_2_-NPs have primarily emerged as a promising component in the development of nano fertilizers which improve plant growth, nutrient uptake, and stress resistance while reducing environmental impact [110,111]. Plants can absorb and utilize nutrients in nano fertilizers more effectively, which in turn increases their nutrient-use efficiency to promote sustainable agricultural practices. CeO_2_-NPs can be used to encapsulate essential nutrients and release them gradually, providing a constant nutrient supply over time and reducing the risk of nutrient leaching and environmental contamination from fertilizer runoff [112].

Some micronutrients, such as Fe, B, Mn, Zn, Cu, Mo, Ni, and Cl, are required in trace concentrations of less than 100 ppm for various physiological processes of plants. Nanoceria modifies the chemical environment of the rhizosphere by regulating pH and redox reactions, which enhances the solubility and thus the bioavailability of essential micronutrients. In their experiment with CeO_2_ nano fertilizer added into cabbage (*Brassica oleracea* var. *capitata* L.) crop as a supplement to NPK fertilizers, Abdulhameed et al. [100] noted a three times increased cabbage head weight over the control plants due to enhanced micronutrient supply to plants. Moreover, they observed a significantly high chlorophyll content in cabbage leaves that received NPK + CeO_2_ nano fertilizer. As noted by Awad et al. [113], foliar spraying of CeO_2_-NPs is particularly useful for micronutrient delivery and promoting photosynthesis.

As discussed by Adhikary and Basak [114], crop seeds primed with CeO_2_-NPs show faster germination and better seedling growth and vigor. Moreover, NPs help protect seeds from oxidative stress during the early stages of germination. For example, polyacrylic-acid-coated nanoceria has been proven to improve salt tolerance in rapeseed and cotton seeds [115]. Similarly, uncoated CeO_2_-NPs at a 500 mg/L dose were reported to significantly alleviate DNA damage in NaCl-treated rice [33]. Gao et al. [89] used alfalfa (*Medicago sativa*) seeds to explore the potential benefits of CeO_2_-NP priming on seed germination and resilience to salt tolerance. They detected significant alleviation of salt stress in alfalfa seeds at concentrations of CeO_2_-NPs up to 500 mg/L, with the 50 mg/L treatment showing the best effect. Moreover, CeO_2_-NPs can also diminish the heavy metal stress in plants by (1) reducing the bioavailability of these metals in the soil, (2) regulating the expression of genes responsible for their transport, (3) strengthening antioxidant systems, or (4) stimulating the secretion of organic acids or metal chelators into the soil [112]. According to Rossi et al. [105], nano-sized Ce^4+^ oxide prevents Cd translocation into soybean (*Glycine max* L.) shoots, probably by chelation of Cd and sequestration in the root cell vacuole. Wang et al. [55] reported that CeO_2_-NPs reduce the inhibition of chlorophyll biosynthesis, resulting from the presence of CdCl_2_ in the hydroponic solution.

CeO_2_-NPs can be used in agriculture to enhance the disease resilience of crops. In particular, the redox activity and antioxidant capabilities of nanoceria contribute to mitigating the effects of pathogens and promoting overall plant health. In their study, Adisa et al. [116] detected an increased fruit dry weight (67%) and lycopene content (9%) in tomato plants infested with *Fusarium* wilt pathogen at the foliar exposure of CeO_2_-NPs at 250 mg/L, compared to the infested and untreated control. They further demonstrated a minimal negative effect of CeO_2_-NPs on the nutritional value of tomato fruit while simultaneously suppressing *Fusarium* wilt disease

The effects of CeO_2_-NPs on plant physiology and productivity are distinct from those of other metal oxide NPs. For instance, ZnO-NPs are known to enhance Zn uptake and boost enzyme activity, but they can be phytotoxic at lower concentrations compared to CeO_2_-NPs. TiO_2_-NPs have shown positive effects on photosynthesis and chlorophyll synthesis, but their inert nature limits the redox interaction-based benefits seen with CeO_2_ [117,118]. CuO-NPs, although antimicrobial, often exhibit higher phytotoxicity due to Cu ion release and oxidative stress [90]. CeO_2_-NPs generally exert biphasic effects—stimulating growth and nutrient uptake at low concentrations due to their ROS-regulating ability, but causing toxicity at higher doses [2,25]. This dose-dependent duality is less pronounced in TiO_2_ and ZnO nanoparticles [7,74]. Moreover, in saline or heavy metal-stressed environments, CeO_2_-NPs outperform many other metal oxide NPs by alleviating stress through antioxidant mechanisms rather than merely modifying nutrient profiles [21,24].

As nanotechnology in agriculture is still emerging, we only have patchy knowledge regarding the impacts of engineered nano materials on soil systems and crop production. Currently, there is no comprehensive study in the literature that evaluates the efficacy and environmental impact of nanoceria agrochemicals under field conditions. This is a crucial knowledge gap, and more work will be necessary for a sound evaluation of the benefits and risks of nano agrochemicals compared to existing products. Further, the economic viability of large-scale production of CeO_2_-NPs for widespread use in agriculture is yet to be assessed. In addition, proper regulatory frameworks need to be established to ensure the safe use of CeO_2_-based nano fertilizers in agriculture [110]. For this purpose, ecotoxicological assessments should be carried out to determine the threshold safe limits of CeO_2_-NPs for various crop species. Long-term and regular monitoring of soil and crop systems where nanoceria is applied will help understand the safe threshold limits under a given set of environmental parameters. Additionally, research studies focusing on the persistence, bioaccumulation, and degradation pathways of CeO_2_-NPs in diverse settings of soil plant systems are required. The authors can further recommend the development of clear and practical guidelines for proper handling, storage, and application of CeO_2_-based nano fertilizers, the introduction of globally recognized testing protocols and standards for nanoceria, and directives for product certification and labeling to improve the safe use of nanoceria in agricultural applications.

## 7. Conclusions and Outlook

CeO_2_-NPs have emerged as a promising tool in modern agriculture due to their unique physicochemical properties, including high redox activity, antioxidant potential, and biocompatibility. Their application in soil–plant systems has demonstrated both beneficial and adverse effects, depending on concentration, particle size, coating, and interactions with plants and soil microbiota. At optimal concentrations, CeO_2_-NPs can significantly enhance plant growth, morphology, antioxidant activity, and biochemical functions. They hold the potential to improve nutrient use efficiency, alleviate oxidative stress, and promote resilience against environmental stressors. Such benefits could contribute to increased agricultural productivity, making CeO_2_-NPs a valuable tool for sustainable farming practices. Additionally, their interaction with soil microbial communities can stimulate beneficial microbes, enhancing soil health and fertility.

However, the adverse effects of CeO_2_-NPs at higher concentrations cannot be disregarded. Excessive doses can induce phytotoxicity, alter plant morphology and physiology, disrupt microbial communities, and impair soil health. Moreover, the potential for bioaccumulation and biomagnification through the food chain raises significant concerns for human health and environmental safety. The persistence of CeO_2_-NPs in the environment and their long-term impacts on ecosystems remain poorly understood to date.

Several factors, such as the particle size, surface charge, and coating of CeO_2_-NPs, influence their uptake, translocation, and accumulation in plants. These variables, along with plant-specific responses, add complexity to understanding their interactions in soil–plant systems. To harness the potential of CeO_2_-NPs for agricultural applications, it is essential to establish safe and effective usage protocols. For this, future research should focus on elucidating the mechanisms behind the uptake, translocation, and accumulation of CeO_2_-NPs in plants, particularly their movement to edible parts. Evaluating the long-term environmental impacts, including persistence, degradation, and effects on soil and plant ecosystems, is also much needed.

The dual role of CeO_2_-NPs—enhancing plant productivity at optimal doses while posing risks at higher concentrations—highlights the need for balanced and well-regulated applications. Optimizing dosage levels tailored to specific crops and soil conditions is required to minimize risks and maximize benefits. Regulatory frameworks must address their safe use, encompassing standardized testing protocols, monitoring systems, and guidelines for their application in agriculture. Such measures will ensure that CeO_2_-NPs can contribute positively to global food security without compromising environmental and human health. In conclusion, CeO_2_-NPs represent a potential breakthrough in agricultural innovation, offering opportunities for sustainable farming and improved crop production. However, their application demands a cautious, science-driven approach to address unresolved concerns and ensure long-term safety. With continued research and careful management, CeO_2_-NPs could play a significant role in shaping the future of agriculture.

## Figures and Tables

**Table 1:** Growth and yield responses of crop plants under nanoceria treatment.

Plant	Application Rate	Growth Media	Exposure Duration	Response	Reference
*Triticum aestivum* L.	0, 125, 250, and 500 mg/kg	Soil media	94 days	Improved plant growth, shoot biomass and grain yield	Rico et al. [97]
*Lactuca sativa* L. var. *longifolia* Lam.	0–2000 mg/L	Sand	21 days	Biomass reduction at dose above 2000 mg/kg	Zhang et al. [37,98]
*Brassica rapa*	0–1000 mg/L	Soil mixture	35 days	Reduced biomass and seed yield	Ma et al. [96]
*Lactuca sativa*	1000 mg/kg	Potting soil	30 days	Impeded plant growth and biomass	Gui et al. [46]
*Hordeum. vulgare*	500 and 1000 mg/kg	Soil	10 days	Reduced number of spikes, tillers, and leaf area	Marchiol et al. [99]
*Solanum lycopersicum* L.	(0.1–10 mg/L)	Potting mix	70 days	Improved fruit yield and plant growth	Wang et al. [81]
*Helianthus annuus* L.	0–800 mg/kg	Potting mix	35 days	Altered biomass, oxidative stress	Tassi et al. [100]
*Cucumis sativus*	0.2, 2, 20, 200 and 2000 mg/L	Hoagland solution	14 days	No phytotoxicity	Ma et al. [67]
*Coriandrum sativum* L.	62.5, 125, 250, 500 mg/kg	Soil	30 days	Promotion of root and shoot growth at 125 mg/kg No effect on dry biomass	Morales et al. [59]
*Cucumis sativus* L.	400, 800 mg/kg	Mixture of sandy soil, sand, and perlite	53 days	No effect on biomass production, shoot length, and leaf area Decrease in fruit weight at 800 mg/kg	Zhao et al. [101]
*Phaseolus vulgaris* L.	62.5, 125, 250, 500 mg/L	Hoagland solution	15 days	Dose-dependent effects on biomass production	Majumdar et al. [102]
*Arabidopsis thaliana* (L.) *Heynh*	100, 200, 500, 1000, 3000 mg/L	Agar plus Murashige & Skoog solution	25 days	Increase in fresh biomass of roots and shoots at <500 mg/L Signs of toxicity at >1000 mg/L	Yang et al. [75]
*Raphanus raphanistrum* subsp. *sativus* (L.)	10 mg/L	25% Hoagland solution	21 days	No effect on dry biomass No apparent adverse effect on the growth and development of plants	Zhang et al. [85]
*Triticum aestivum* (L.)	100, 500, 1000, 2000 mg/L	25% Hoagland solution	20 days	Increased fresh biomass of roots and shoots at 100 and 500 mg/L; reduced at 1000 and 2000 mg/L Increase in shoot growth at 500 mg/L Decreased root elongation at 100, 1000, and 2000 mg/L	Abbas et al. [83]
*Glycine max* (L.)	100 mg/kg	Soil under different moisture conditions	21 days	Increased fresh and dry biomass of roots and shoots	Cao et al. [103]
*Lathyrus oleraceus* Lam.	100, 200, 500 mg/L	Hoagland solution	12 days	Decreased fresh biomass of roots and shoots at 500 mg/L No effect on dry biomass	Skiba et al. [84] and Skiba et al. [104]
